# A Simple and Versatile Cell-Free Expression Method for Producing Secondary Metabolites

**DOI:** 10.1021/acssynbio.5c00497

**Published:** 2025-12-25

**Authors:** Jaime Lorenzo N. Dinglasan, Namil Lee, Nam Ngoc Pham, Meghana Faltane, Marie Lynde, Katherine B. Louie, Sangeeta Nath, Jay D. Keasling, Hiroshi Otani, Nigel J. Mouncey

**Affiliations:** † US Department of Energy Joint Genome Institute, 1666 Lawrence Berkeley National Laboratory, Berkeley, California 94720, United States; ‡ California Institute for Quantitative Biosciences (QB3), University of California, Berkeley, California 94720, United States; § Joint Bioenergy Institute, Emeryville, California 94608, United States; ∥ Biological Systems and Engineering Division, Lawrence Berkeley National Laboratory, Berkeley, California 94720, United States; ⊥ Environmental Genomics and Systems Biology Division, Lawrence Berkeley National Laboratory, Berkeley, California 94720, United States; # Center for Advanced Bioenergy and Bioproducts Innovation, Lawrence Berkeley National Laboratory, Berkeley, California 94720, United States; ∇ Department of Chemical & Biomolecular Engineering, University of California, Berkeley, California 94720, United States; ○ Department of Bioengineering, University of California, Berkeley, California 94720, United States; ◆ Novo Nordisk Foundation Center for Biosustainability, Technical University of Denmark, Lyngby 2800, Denmark

**Keywords:** secondary metabolites, cell-free expression, natural product synthesis, Streptomyces, type I polyketide synthase

## Abstract

Secondary metabolites are a major source of natural products with industrially relevant bioactivities. Lysate-based cell-free expression (CFE) is an emerging platform for accelerating the discovery and engineering of these natural products. While *Escherichia coli* cell extracts are widely used for CFE, *Streptomyces* extracts are likely to offer a more biochemically compatible environment for their expression. However, current *Streptomyces*-based CFE systems remain underdeveloped, with protocols that are either strain-specific or not readily scalable. To address these limitations and enable broader access to cell-free natural product biosynthesis, we present a generalizable and simple set of reaction conditions that support high-yield protein expression (180–230 μg/mL) in lysates derived from *Streptomyces venezuelae* NRRL B-65422 and *Streptomyces lividans* TK24. Like *E. coli*-based systems, these extracts enable iterative and pathway-level biosynthesis, as demonstrated by the production of the polyketide flaviolin and the cyclic dipeptide albonoursin. Notably, the *S. lividans* lysate outperforms the *E. coli* systems by also supporting the expression and catalytic activity of a (∼250 kDa) type I polyketide synthase (T1PKS), producing its corresponding ethyl ketone product, 2-methyl-3-pentanone, without the need for precursor or post-translational modification supplements. To our knowledge, this represents the first demonstration coupling both expression and catalysis of a megasynthase in a *Streptomyces*-based system, and of a T1PKS in any bacterial extract. By addressing key challenges in the generalizability and scalability of prior *Streptomyces* CFE, we establish a protocol that enables parallelized evaluation of diverse lysate systems and provides a foundation for high-throughput T1PKS engineering *in vitro*.

## Graphical abstract

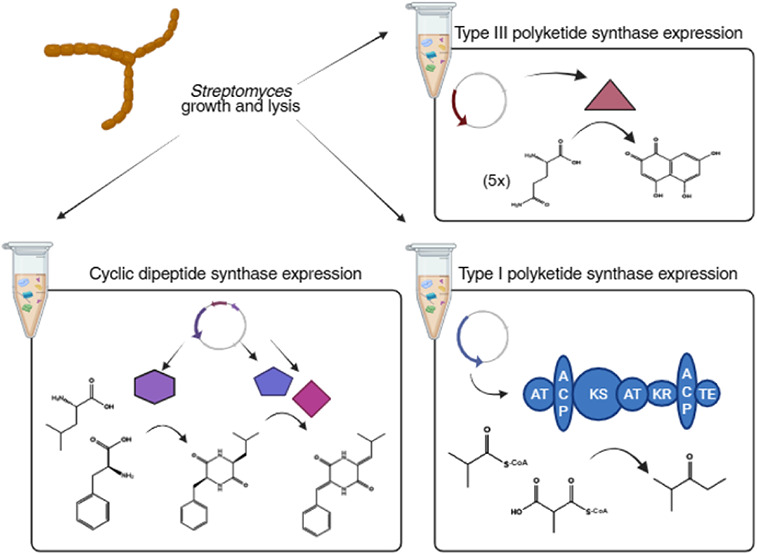

## Introduction

Secondary metabolites possess bioactivities that have historically been most valued in medicine (e.g., antibiotics, antiparasitic, and anti-inflammatory agents)^1,2^ and agriculture (e.g., pesticides).^3^ They are also increasingly considered as nontoxic, environmentally friendly alternatives to petrochemicals used in food safety,^4^ cosmetics,^5^ fuels,^6^ and plastic production.^7^ These natural products can be made microbially, especially in actinomycetes like *Streptomyces*, and cover a wide range of compound classes that reflect the diversity of microbial metabolism.^8–10^ Polyketides, for example, have been studied for decades as therapeutics,^11^ while newer classes, like cyclic dipeptides,^12^ are emerging, highlighting the ongoing discovery of novel chemistries with potential value.^13^ These compounds are typically synthesized by enzymes encoded in the same genomic regions forming biosynthetic gene clusters (BGCs), including biosynthetic and tailoring enzymes.^13^ Engineering these enzymes and their pathways additionally offers access to metabolites with unique or enhanced activities, expanding the portfolio of natural products with potentially beneficial chemistries.^14–18^ In particular, type I polyketide synthases (T1PKSs) responsible for polyketide synthesis are intriguing engineering targets because the multimodular, multidomain architectures of these megasynthases offer unique opportunities for the combinatorial synthesis of new-to-nature compounds.^14^

Advances in genome mining and AI-driven enzyme engineering are rapidly expanding access to a vast and largely untapped natural product space.^10^ Yet, many of these biosynthetic pathways remain empirically uncharacterized due to challenges in expressing and characterizing them in both heterologous and native microbial hosts.^10,19^ Even with automation, transforming and expressing genes in microbes—especially in relatively slow-growing *Streptomyces*—can take several days to weeks without guarantee of proper enzyme production.^20^ Detection and characterization of enzymatic reactions involve tedious processes including protein purification.^21^ As a result, optimizing microbial hosts for natural product synthesis can require months of iterating through design-build-test-learn (DBTL) cycles, and mobilizing them for high-throughput screening (i.e., in enzyme engineering workflows) remains time-consuming and labor-intensive.^22^

Lysate-based cell-free expression (CFE) systems offer a powerful alternative for addressing this bottleneck.^10^ These platforms enable protein synthesis *in vitro* by combining cell extracts containing endogenous transcriptional-translational (TX-TL) and metabolic machinery with (1) a buffered mix of reaction components (e.g., dNTPs, amino acids, cofactors, salts) that energize TX-TL and metabolism, hereafter referred to as “energy mix”, and (2) DNA template(s) encoding the protein(s)-of-interest.^23^ By decoupling expression from growth, cell-free systems enable enzyme production and product formation within hours.^22^ Their open format bypasses the need for transformation, allows direct product detection from crude lysates, and is inherently suited to miniaturization, automation, and rapid optimization.^24^ As such, cell-free platforms are emerging as versatile tools for accelerating natural product discovery and engineering.^25–28^ Bacterial extracts have been applied to synthesize compounds from various natural product classes including RiPPs,^29–31^ terpenoids,^32^ a small polyketide,^33^ and nonribosomal peptides^34,35^ like cyclic dipeptides.^36,37^

To date, most cell-free platforms used for studying natural product biosynthesis have relied on *Escherichia coli* lysates.^23^ While these are well-characterized and inexpensive, they can fall short when expressing GC-rich biosynthetic genes from actinomycetes, requiring codon optimization, and may result in poor protein solubility and incomplete translation and post-translational modification.^26,33,35,38^ In particular, reports of expressing complex functional T1PKSs through *E. coli* lysate-based CFE are scarce and lack evidence of successful catalysis.^27^ These extracts require the coexpression of a heterologous phosphopantetheinyl transferase (PPTase) to activate these megasynthases,^35,37^ and have reportedly resulted in the truncated synthesis of a large (>200 kDa) module of a T1PKS.^39^ In contrast, cell-free systems derived from *Streptomyces* cells, where such megasynthases are routinely produced and activated, more closely resemble the native biochemical environment of these enzymes.^28,40,41^ They can accommodate native gene sequences, support endogenous post-translational modifications, and thus, potentially improve solubility and catalytic activity of complex biosynthetic proteins.^28^ Further, aligning the expression and screening environment with the final production host minimizes context-dependent failures when moving promising constructs into *Streptomyces* strains for scale-up. This consideration is critical, given that *Streptomyces* strains remain the industrial standard for producing natural products from complex enzymes, like PKSs, and BGCs, presumably due to their biochemical compatibility and product secretion capabilities.^42–44^

Despite these advantages, only two *Streptomyces* CFE platforms have been developed and applied to natural product biosynthesis.^28^ However, the protocols for generating these are either strain-specific or require purified enzyme supplements, constraining their generalizability and scalability. Additionally, these foundational studies focused on protein yields across *Streptomyces* and well-established *E. coli* extracts without comparing differences in their abilities to support complex natural product biosynthesis. Here, we expand methods for generating efficient actinomycete-based cell-free systems by defining a set of conditions that allow up to 185 and 230 μg/mL protein yields in *Streptomyces venezuelae* NRRL B-65422 and *Streptomyces lividans* TK24 lysates, respectively. Our protocol can be applied to produce proteins in non-*Streptomyces* actinomycetes and does not require the time-consuming purification of protein additives, enabling the rapid testing of various expression environments in parallel. We demonstrate that these systems can produce biosynthetic enzymes and generate secondary metabolites *in vitro*, and we uniquely compare these capabilities to that of an *E. coli*-based system. We further demonstrate the superior performance of the minimally optimized *S. lividans* for producing a functional T1PKS and its cognate product, 2-methyl-3-pentanone. By establishing these platforms, we build on the foundation for rapid, scalable workflows to discover and engineer natural products with high throughput.

## Results and Discussion

### Optimization of a Procedure for Versatile and Scalable CFE in *Streptomyces*

We evaluated two *Streptomyces* lysate environments—derived from *S. venezuelae* NRRL B-65422 and *S. lividans* TK24—for their ability to support natural product synthesis. They were selected due to their extensive use in natural product synthesis^43^ and genetic tractability,^45,46^ and because protocols for preparing active cell-free extracts had previously been reported for closely related strains: *S. venezuelae* ATCC 10712 ^47^ and *S. lividans* B-12275.^38^ *S. venezuelae* NRRL B-65422 is an emerging model strain commonly used in various research groups and shares 99.99% genomic identity to ATCC 10712.^46,48,49^ This strain similarly has a shorter doubling time (ca. 40 min) and forms smaller mycelial clumps in liquid media than typical *Streptomyces* strains, making it suitable for rapid and standardized lysate preparation.^41^ Notably, the previously reported *S. lividans* B-12275-based system required three purified translation factors and a supplemented pyruvate kinase to achieve high yields,^50^ which would limit scalability for high-throughput natural product discovery and engineering efforts. Protein purification introduces a significant bottleneck to scale, as it requires time- and labor-intensive steps such as expression, lysis, chromatography, and buffer exchange for each individual protein. These procedures are difficult to automate and are subject to batch-to-batch variability. We therefore selected *S. lividans* TK24 with the aim of establishing an industrially relevant *S. lividans* based CFE platform. TK24 is a widely used heterologous expression host with low endogenous protease activity and robust genetic tools.^43,51,52^ We sought to leverage these advantages for potentially building a robust yet simplified system by not relying on purified protein additives. Although CFE using a *S. lividans* TK24 cell lysate was previously reported, the lysate preparation protocol was not described.^53^ Nonetheless, the extract reportedly produced ∼46 kDa *Streptomyces* enzymes with increased solubility compared to an *E. coli* extract, providing further basis for its optimization for natural product synthesis.

We initially attempted to reproduce Moore et al.’s^54^ and Li et al.’s^40^ protocols for preparing active *S. venezuelae* and *S. lividans* extracts, respectively. Protein production was assessed by fluorescence readouts from the pTU1-A-SP44-*sfgfp* construct previously developed by Moore and colleagues, which carries a *Streptomyces* codon optimized *sfgfp* sequence expressed from the artificial SP44 promoter.^47^ Moore et al.’s protocol involved lysing *S. venezuelae* ATCC 10712 cells grown in GYM media and activating the extracts with a “minimal” energy mix tailored specifically to ATCC 10712.^54^ Specifically, this formula is a simplified version of the energy mixes originally developed for *E. coli*, containing cofactors and additives like NAD+, CoA, folinic acid, and spermidine. However, when we attempted to apply their growth and lysis protocol in combination with their minimal energy mix formula, no sfGFP signal was detected using the cell lysate from strains NRRL B-65422 or TK24. This outcome may reflect the sensitivity of minimal formulations to variations in extract composition, which can arise from both strain-level and procedural differences. Omitting multiple cofactors and additives from the energy mix makes the reaction environment more dependent on the endogenous metabolite composition of the lysate. Such composition can differ across strains due to physiological factors. Even for NRRL B-65422 and ATCC 10712, which are nearly genetically identical, documented differences in pigment biosynthesis,^46^ stress responses,^55^ and plasmid content^46^ may influence reaction performance and alter the abundance of key metabolites and enzymes supporting transcription–translation. At the same time, even subtle procedural differences during extract preparation, such as variations in sonication intensity or lysis efficiency, can further shift metabolite and cofactor levels, compounding this sensitivity. For example, Moore et al. recommended adjusting sonication energy based on extract color or homogeneity (approximately 1200 J per 5 mL suspension). To minimize variability, we applied a uniform energy input of 1200 J per 5 mL suspension for both strains. We reason that formulations with greater biochemical redundancy are more robust across laboratories and strains. Consequently, we prioritized developing a more complex yet generalizable formulation capable of sustaining protein synthesis in diverse *Streptomyces* extracts rather than reoptimizing minimal, strain-specific systems.

We next applied the methods of Li and coworkers, such as growth in YEME media^38^ and the sonication lysis parameters.^40^ We then supplemented reactions with the PANOx-SP energy mix^56^ originally developed for *E. coli*, which shares its core composition with Li’s mix but lacks the purified pyruvate kinase for boosting energy regeneration.^38^ Using these conditions, we detected measurable sfGFP production from both *S. venezuelae* and *S. lividans* extracts, consistent with Li’s report of cross-*Streptomyces* strain compatibility. Initial yields of 1.71 μg/mL and 0.765 μg/mL, respectively, were lower than those reported by Li et al. (Supplemental Figure 1), likely due to the absence of pyruvate kinase.

Taken together, these observations suggest that formulations incorporating a broader set of cofactors and energy substrates are more robust across strains and laboratories than minimal formulations. We therefore proceeded with optimization using this less minimal, more biochemically complete energy mix as a foundation for developing a generalizable *Streptomyces* cell-free system. The only exception was pyruvate kinase, which we deliberately omitted due to its cost as a purified enzyme supplement. Instead, we focused on optimizing growth, lysis, and reaction parameters to enhance expression.

We first focused on optimizing CFE conditions for *S. venezuelae* NRRL B-65422 extracts due to the strain’s ease of culture monitoring by optical density and shorter time-to-harvest.^41,46^ Different growth media and lysis sonication energies (i.e., joules delivered) were first compared (Figure 1A,B). Briefly, cells were grown in either GYM or YEME media. After resuspension in 0.8 mL prelysis buffer per gram cell weight, 250 μL aliquots were sonicated by applying 75, 150, or 225 J. The generated extracts were then combined with the PANOx-SP energy mix and pTU1-A-SP44-*sfgfp*, and fluorescence was measured after 4 h. sfGFP signal was detected in the NRRL B-65422 lysates prepared only from cells grown in YEME, reinforcing that growth of this strain in GYM media does not yield active extracts, even in combination with the less minimal PANOx-SP energy mix. Yields of sfGFP approached ∼2.5 μg/mL when 150 J per 250 μL cell resuspension were applied to generate lysates. The total protein concentration of this extract was ∼20 μg/mL and could be increased to 30 μg/mL by reducing the volume of the resuspension buffer from 0.8 to 0.4 mL per gram cell weight. Using extracts with higher total protein content increases the concentration of the TX-TL machinery in the CFE reaction, facilitating enhanced protein synthesis.^40^ Indeed, using the more concentrated extract improved sfGFP yield to ∼4 μg/mL (Figure 1C).

**Figure 1:**
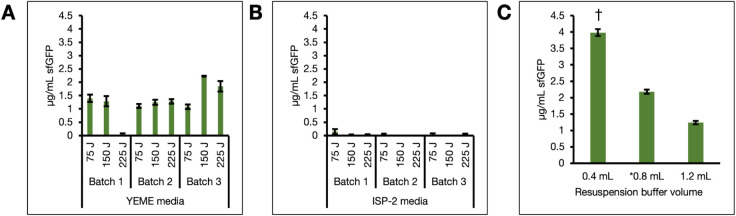
Optimization of growth and lysis conditions for *S. venezuelae* CFE. Batches of cells (*n* = 3) were grown in (A) YEME or (B) GYM media then resuspended in 0.8 mL lysis buffer per gram cell weight. Per cell batch, aliquots of 250 μL cell resuspension (*n* = 3) were lysed with a sonicator by delivering 75, 150, or 225 Joules. The resulting lysates were used in CFE reactions. (C) Varying volumes of resuspension buffer (mL per gram cell weight) were used to resuspend cells grown in YEME for lysis (*n* = 3). CFE reactions were then assembled by combining consistent volumes of each extract with the plasmid and energy mix. All reactions were prepared using the pTU1-A-SP44-*sfgfp* plasmid and *E. coli* PANOx-SP energy mix. Fluorescence was measured after incubation at 30 °C for 4 h. Error bars represent the standard error of the mean. The asterisk (*) is used to designate the initially applied condition and the dagger (†) denotes a significant difference (*p* < 0.005, Student’s *t*-test) between the optimized and previously applied condition in C).

The inability of GYM-grown cells to generate active extracts, despite prior reports of its use in other *Streptomyces* CFE systems, likely reflects differences in the physicochemical environments established by the two media. The high sucrose concentration in YEME (∼340 g L^– 1^) imposes osmotic stress that reduces mycelial aggregation and promotes more dispersed growth among *Streptomyces*,^57^ which may form more homogeneous cells, facilitate more uniform lysis and yield lysates with better access to soluble, active cellular components. Moreover, YEME is prepared with additional supplements such as peptone and MgCl_2_·6H_2_O that provide extra nitrogen sources and divalent cations known to influence protein synthesis and enzyme stability. The richer formulation of YEME compared to GYM likely makes it a more robust and forgiving medium for establishing a new cell-free protein synthesis system, especially when strain-specific requirements have not yet been optimized at the reaction level. Although further fine-tuning of ionic composition or nitrogen sources in the energy mix might have improved expression from GYM-derived extracts, our objective was to first identify growth conditions that reliably produced active lysates before optimizing downstream reaction chemistry. YEME fulfilled this criterion and was therefore selected for all subsequent experiments.

With confirmation of protein production in these extracts, the TX-TL machinery could be better energized by modifying components of the energy mix. Because the PANOx-SP energy mix designed for *E. coli* CFE still outperformed the previously published *S. venezuelae* “minimal mix”^47^ (Figure 2A) under our optimized growth and lysis conditions, we proceeded to modify the former. We first modified the reaction’s energy substrates, which are metabolites that regenerate ATP in the lysate to drive TX-TL^58^ (Figure 2B). The PANOx-SP system utilizes a combination of phosphoenolpyruvate (PEP) and oxalic acid, where the oxidation of PEP to pyruvate releases ATP and oxalic acid serves to inhibit the reverse reaction. However, oxalic acid potentially impairs protein yields in non-*E. coli* lysates.^58,59^ Indeed, omission of oxalic acid yielded a higher GFP signal (Figure 2B). 3-phosphoglycerate (3-PGA) is an alternative energy source that lies upstream of PEP in the glycolytic pathway and is thus eventually oxidized to pyruvate to generate ATP as well. It is reportedly more stable in diverse bacterial extracts including *S. venezuelae*.^47,60^ Moore et al. used 3-PGA in combination with glucose-6-phosphate, which inhibits ATP consuming steps in the top half of glycolysis, as a preferred system in *S. venezuelae* extracts.^47^ This ATP-regenerating system is similarly superior to the PEP in our lysate, especially in the absence of oxalic acid (Figure 2B).

**Figure 2:**
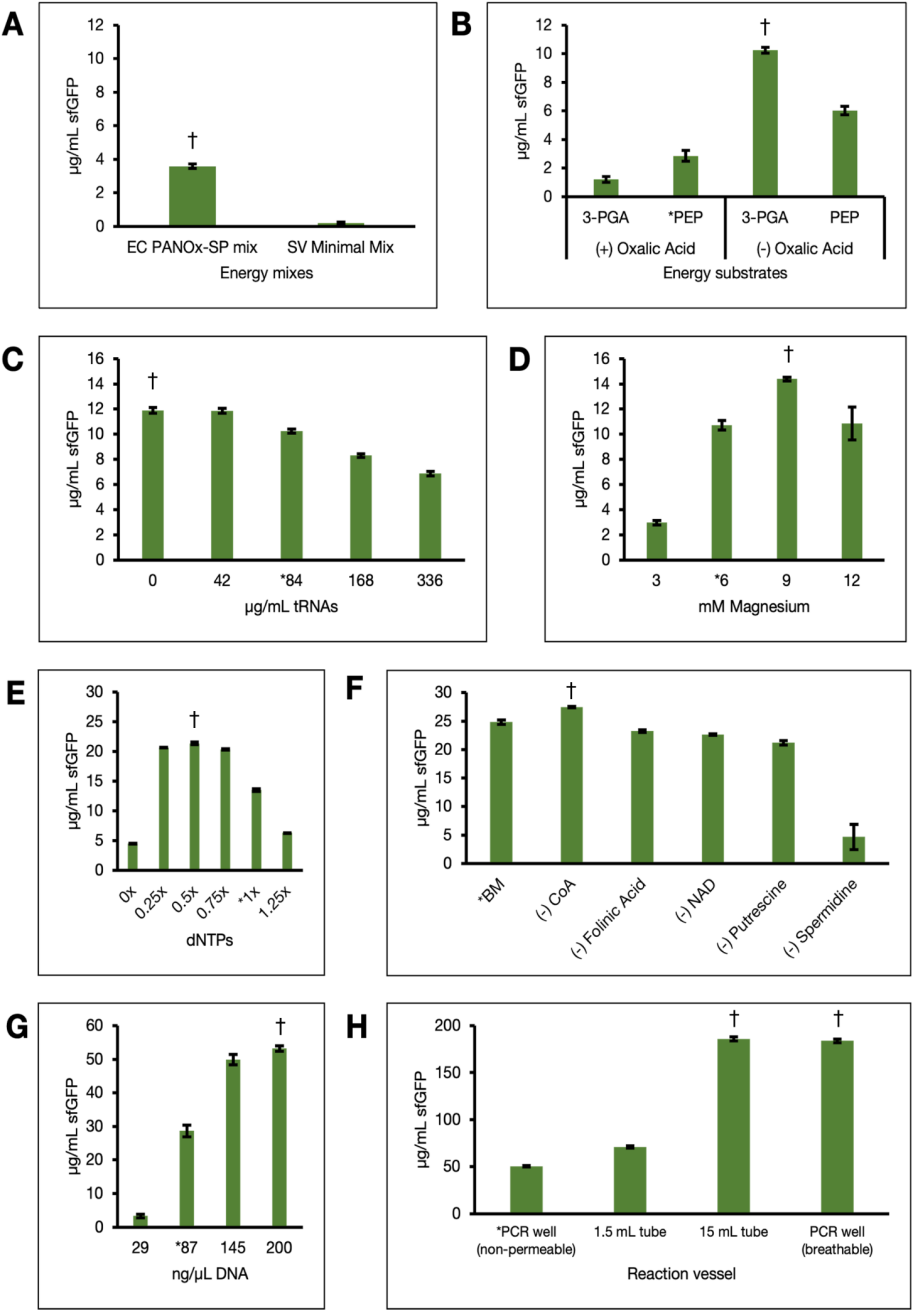
Sequential optimization of reaction conditions for *S. venezuelae* CFE. Following optimized growth and lysis conditions, *S. venezuelae* lysates were used to prepare CFE reactions with varying reaction parameters. (A) Comparison of fluorescence readouts between the PANOx-SP energy mix previously optimized for *E. coli* (EC) extracts and the Minimal Mix optimized by Moore et al. for *S. venezuelae* (SV) lysate expression. (B) Evaluation of different primary and secondary energy substrates for ATP regeneration. (C) Assessment of different concentrations of exogenously supplied tRNAs. (D) Identification of an optimal magnesium concentration. (E) Optimization of dinucleotide triphosphate (ATP, GTP, CTP, and UTP) concentrations. The 1× condition uses 1.2 mM ATP, 1.2 mM GTP, 0.68 mM CTP, and 0.68 mM UTP. (F) Singular omission of additives from the PANOx-SP mix to assess their individual impact on CFE. (G) Enhancement of sfGFP yields by increasing the starting concentration of template DNA. (H) Significant improvement of *Streptomyces* TX-TL by testing different reaction vessels to improve oxygen availability. Asterisks (*) are used to designate the initial condition for each round of optimization and daggers (†) denote a significant difference (*p* < 0.005, Student’s *t*-test) between the optimal and previously applied conditions. Error bars represent the standard error of the mean (*n* = 3).

We found that exogenous *E. coli*-sourced tRNAs, which were initially added to the reactions at 84 μg/mL, are actually deleterious to TX-TL in our system (Figure 2C). This is inconsistent with the reported positive and neutral impacts of this component on lysates derived from *S. lividans* B-12275 and *S. venezuelae* ATCC 10712, respectively.^47,50^ Optimizing magnesium and dNTP concentrations led to moderate gains (Figure 2D,E). These metabolites directly impact transcription and translation but, as cofactors, these components are known to interact in complex ways with each other and with other enzymes and metabolites in the reaction, and can cause cofactor imbalances if present in excessive amounts.^58,61,62^ For example, dNTPs can chelate free magnesium ions.^63^ Consistent with the results from Moore et al., we found that removing certain supplements (i.e., CoA, NAD, folinic acid, putrescine) used in *E. coli* CFE formulations also minimally impacted *Streptomyces* CFE^47^ with the exception of spermidine, a polyamine that stabilizes mRNA^58^ (Figure 2F). Spermidine is an essential component of our energy mix presumably because our lysates have insufficient endogenous levels of this metabolite, supporting our observation that differentially prepared lysates can benefit from more complex energy mix compositions. However, increasing spermidine concentrations had no substantial positive impact on the sfGFP yield (Supplemental Figure 2). CoA removal slightly improved CFE and, hence, was removed from the formulation. CoA can counteract ATP and NADH formation when present in excess, by blocking glycolysis or driving ATP-consuming reactions (e.g., acetyl-CoA synthesis). CoA is at the core of *Streptomyces* primary and secondary metabolism, so these lysates may already carry sufficient endogenous CoA or have the capacity to regenerate it through acyl-CoA turnover.^28^ While they had little negative impact on *S. venezuelae* CFE, we retained NAD, folinic acid, and putrescine in the mix in case these were necessary for CFE in the *S. lividans* extract.

Following optimization of the energy mix composition, increasing the DNA template concentration from 87 ng/μL to 200 ng/μL improved sfGFP yields from approximately 28 μg/mL to 54 μg/mL (Figure 2G). This yield was on par with initial iterations of the aforementioned *Streptomyces* systems, which were able to sufficiently synthesize proteins ranging from 12 to 70 kDa in size.^38,41^ We sought to improve the efficiency of our system further given our interest in synthesizing much larger enzymes, but aimed to do so without complicating our protocol. Xu et al. reported GFP yields of approximately 400 μg/mL using *S. lividans* B-12275 extracts supplemented with three purified translation-related factors, suggesting that the native translational machinery in *Streptomyces* lysates may be a key limiting step in protein synthesis.^50^ While similar supplementation could potentially enhance expression in our system, purification would reduce the simplicity and scalability needed for high-throughput applications in natural product studies, such as gene discovery or variant screening. Instead, we focused on identifying process-level parameters that enhance translation without requiring exogenous protein supplementation.

Oxygenation is known to drive metabolism and TX-TL in *E. coli* extracts^61,64^ and we hypothesized that this might be especially limiting in our system given that *Streptomyces* are obligate aerobes.^65^ So far, we had been running our reactions in 10 μL volumes within wells of a 96-well PCR plate sealed with a nonpermeable film. To assess whether a larger headspace could enhance *Streptomyces* extract performance, we compared these to 30 μL reactions incubated in 1.5 mL tubes and 15 mL conical tubes for 4 h. As expected, this change led to a significant ∼4-fold increase in protein yield (Figure 2H). Similar yields are observed from 10 μL reactions in wells of a PCR plate sealed with a breathable membrane and incubated in a humid chamber to ensure no evaporation from the sample over 4 h, confirming that these improvements are not dependent on the geometry of the reaction vessel^61^ (Figure 2H). Together, these data suggest that aeration is a tunable parameter when boosting cell-free expression from *Streptomyces* lysates, as prepared here. In contrast, Gamboa-Suasnavart and colleagues noted that aeration of their *S. venezuelae* ATCC 10712 extracts had no effect on TX-TL. Our observations are likely attributed to the high concentration of sucrose (34% (w/v)) in the YEME medium, which could reduce oxygen transfer rates in *Streptomyces* cultures.^66^ Consequently, the metabolism in our extracts may be primed with a higher reliance on oxygen availability, resulting in significant expression improvements with aeration. However, this requires further investigation to determine whether the oxygen effect reflects the presence of electron transport chain (ETC) enzymes bound to residual membrane vesicles in the lysate. These enzymes would in turn enable oxidative phosphorylation or link NAD+/NADH balance to oxygenation as observed in *E. coli* extracts.^61^ This warrants future mechanistic studies such as utilizing ETC inhibitors to investigate the membrane fraction of the extracts.

Subsequently, we compared the sfGFP production using cell lysates from various organisms (Figure 3). At first, we compared the sfGFP yields from *S. venezuelae*, *S. lividans* and *E. coli* lysates after 16 h of the cell-free reaction (Figure 3A). Our *S. venezuelae* extract produced 185.47 ± 3.48 μg/mL sfGFP. We applied the same growth, lysis, and reaction conditions to our *S. lividans* TK24 extract which yielded even higher sfGFP titers, reaching approximately 228.14 ± 16.12 μg/mL (Figure 3A). This result validates the TK24’s robust transcription-translation activity and supports our hypothesis that this strain, as a widely used heterologous host, could serve as a strong chassis for CFE without requiring purified protein supplements. Notably, reaction yields for both extracts plateau within the first 5 h, regardless of oxygen availability (Figure 3B,C). The plasmid and reaction conditions described here also enabled sfGFP production in *E. coli* extracts (Figure 3A–C), which was unsurprising since constitutive expression from SP44 in *E. coli* has been reported^54^ and the current master mix retains components that are necessary for CFE in these lysates. While these conditions are not optimal for *E. coli* lysates, they enable the parallel testing of biosynthetic genes in different expression contexts (*E. coli* vs *Streptomyces*) from the same set of constructs and with minimal reagent preparation.

**Figure 3:**
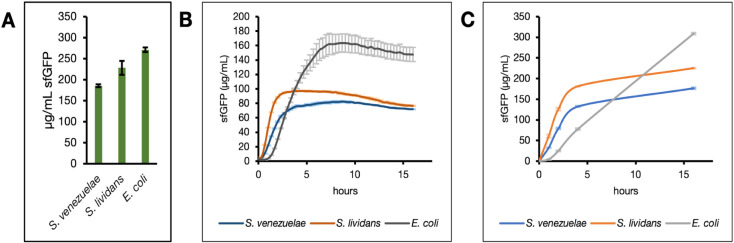
Applying conditions optimized for *S. venezeulae* extracts to *S. lividans* and *E. coli*lysates. (A) sfGFP yields from reactions incubated for 16 h in 15 mL conical tubes. Asterisks (*) are used to designate the initial condition for each round of optimization and daggers (†) denote a significant difference (*p* < 0.005, Student’s *t*-test) between the optimal and previously applied conditions. Dynamics of sfGFP expression in reactions run in (B) wells of a sealed PCR plate and (C) 15 mL conical tubes. Error bars represent the standard error of the mean (*n* = 3).

These conditions additionally enable expression in extracts of non-*Streptomyces* actinomycetes—*Saccharopolyspora erythraea* NRRL 2338, and *Rhodococcus jostii* RHA1—within hours, albeit at lower yields (<100 μg/mL), further demonstrating the generalizability of this protocol (Supplemental Figure 3). *S. erythraea* is a producer of clinically used antibiotic, erythromycin,^67^ while *R. jostii* is known for its metabolic capability to convert plant-derived compounds into industry-relevant compounds such as triacylglycerol.^68^ Further optimization of growth and lysis conditions for these genera will likely improve yields from their extracts and enable rapid characterization of diverse metabolic pathways. The common set of conditions for CFE across these different actinomycete extracts effectively enables mobilizing various expression environments to test BGC expression, which may improve success rates of secondary metabolite production from cloned BGCs.^10^

The yields from *S. venezuelae* and *S. lividans* lysates reported above were the highest sfGFP titers we achieved across different batches of these extracts. From another set of independently generated lysates prepared using our optimized conditions, we saw nearly identical sfGFP titers with *S. venezuelae* producing 176.28 ± 3.48 μg/mL and *S. lividans* producing 220.84 ± 0.84 μg/mL. Meanwhile, the poorest performing batches of extracts produced 159.52 ± 1.95 μg/mL and 169 ± 3.97 μg/mL sfGFP, respectively. While further improvements in sfGFP yield were likely achievable through additional tuning of reaction parameters, our aim was not to maximize reporter expression. Instead, our goal was to achieve sufficient protein synthesis to support product detection from the one-pot expression and catalytic activity of complex biosynthetic enzymes. At ∼180–230 μg/mL, our highest-yielding *S. venezuelae* and *S. lividans* systems already compare favorably to heterologous expression levels typically reported in *Streptomyces* cells, whose expression ranges from 10 to 300 mg/L for similarly sized proteins.^52^ Moreover, the *S. venezuelae* ATCC 10712 lysate developed by Moore et al. that produced up to 174 μg/mL sfGFP from the same construct used here was also able to produce larger biosynthetic enzymes: an uncharacterized NRPS from *S.* rimosus (110 kDa) and the TxtB NRPS from *S. scabiei* (162 kDa).^47,54^ These results laid the foundation for testing the production of functional biosynthetic enzymes using our top-performing extracts, including large, multidomain T1PKSs.

### Validating Secondary Metabolite Synthesis in *Streptomyces* Cell-Free Extracts

To evaluate the capacity of our extracts to support iterative biosynthetic enzyme activity as well as functional multienzyme pathways, we expressed RppA from *Streptomyces griseus* and the albonoursin BGC from *Streptomyces noursei*. RppA is a type III polyketide synthase (T3PKS) that catalyzes the iterative condensation of malonyl-CoA units to form the polyketide intermediate 1,3,6,8-tetrahydroxynaphthalene (THN), which spontaneously oxidizes into the pigmented compound flaviolin.^69^ Albonoursin is a cyclic dipeptide whose biosynthesis involves the condensation of l-Phenylalanine (l-Phe) and l-Leucine (l-Leu) from amino acyl tRNA catalyzed by a cyclic dipeptide synthase AlbC, followed by its oxidation by enzymes encoded by *albA* and *albB*.^70^ Favorably, flaviolin and albonoursin production can be rapidly monitored in real time by measuring absorbance at 340 and 318 nm, respectively. Native sequences of *rppA* and the albonoursin biosynthetic gene cluster were cloned into pTU1-A-SP44. Expression from these plasmids was first confirmed in the *E. coli* extract given the previous detection of the flaviolin product in *E. coli* CFE reactions^33^ and the report of heterologous albonoursin production in *E. coli* cells.^70^ Time-course measurements confirmed flaviolin and albonoursin production in the *E. coli* extract (Figure 4A,B). It should be noted that these reactions were run in a sealed 96-well plate due to significant evaporation from aerated wells within the plate reader beyond 3 h, which precluded overnight time-course measurements. To orthogonally validate their production of flaviolin and albonoursin in these reactions, they were detected via LC-MS (Figure 4C,D) and MS/MS spectra were used to confirm the identity of these metabolites in the reactions (Supplemental Figure 4).

**Figure 4:**
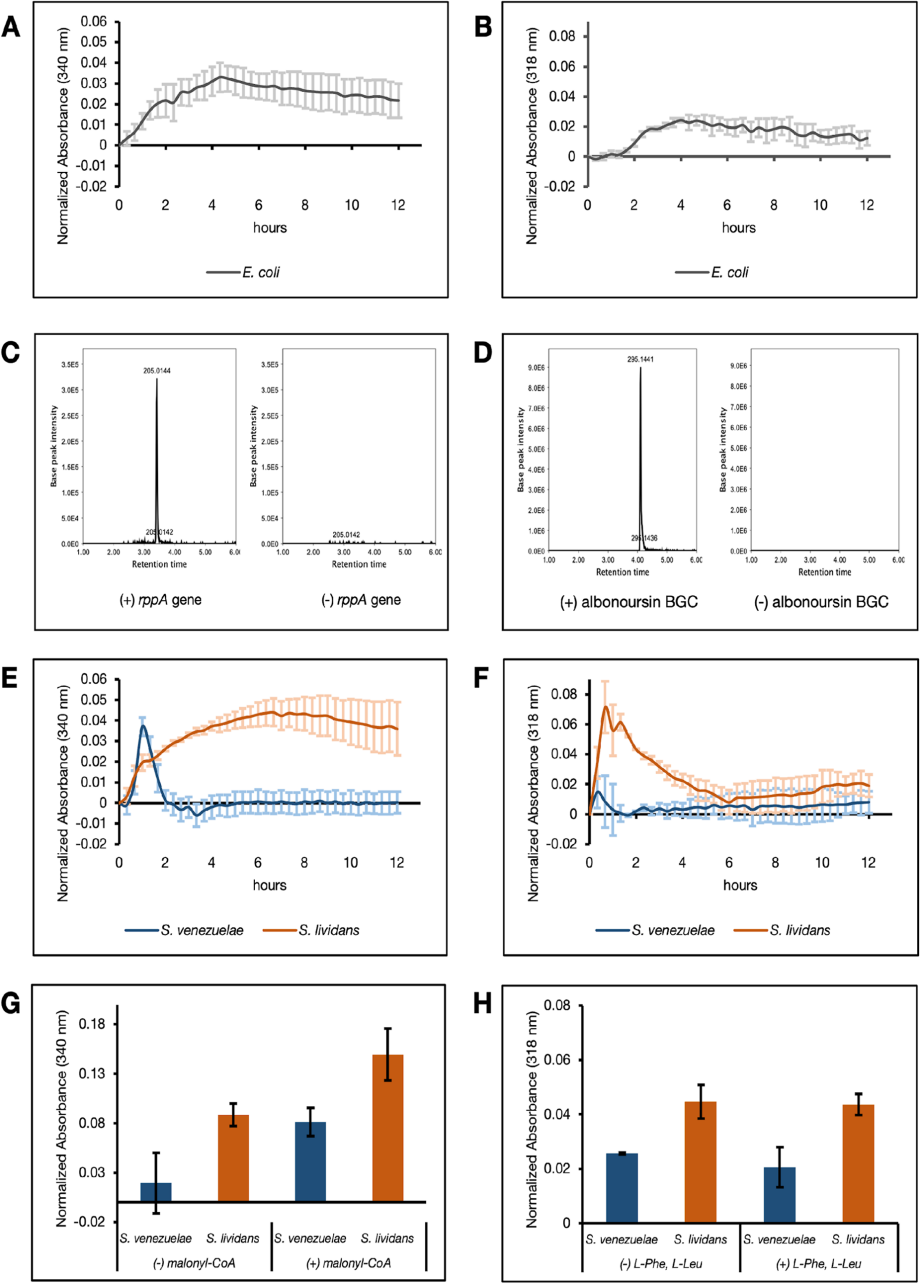
Evaluating the synthesis of flaviolin and albonoursin in *E. coli*, *S. venezuelae*, and *S. lividans* extracts. Genes responsible for flaviolin and albonoursin synthesis were cloned into the pTU1-A-SP44 vector then added to CFE reactions (*n* = 3). All absorbance measurements from CFE reactions initiated with a plasmid were normalized to control reactions initiated without the plasmid, and to time-zero. (A) Verification of flaviolin synthesis from the pTU1-A-SP44-*rppA* construct in *E. coli* lysates over 12 h. (B) Verification of albonoursin synthesis from the pTU1-A-SP44-*albABC* construct in *E. coli* lysates over 12 h. (C, D) Validation of (C) flaviolin and (D) albonoursin production in the *E. coli* extract using LC-MS/M*S.* Peaks corresponding to the (C) flaviolin precursor *m*/*z* ([M-H]^−^) of 205.014 and (D) albonoursin precursor *m*/*z* ([M+K]^+^) of 295.144 are only identified in Extracted Ion Chromatograms from the *E. coli* extract-based reactions initiated with plasmid, and not from control reactions initiated with water. (E, F) Normalized time-course measurements of (E) flaviolin and (F) albonoursin synthesis in CFE reactions prepared with *Streptomyces* extracts. (G) Normalized end-point absorbance measurements from *Streptomyces* extract-based reactions incubated in 15 mL vessels overnight (16 h) with or without 1 mM malonyl-CoA. (H) Normalized end-point absorbance measurements from *Streptomyces* extract-based reactions incubated in 15 mL vessels overnight (16 h) with or without additional 0.75 mM l-Phe and 0.75 mM l-Leu. Daggers (†) indicate a significant difference (*p* < 0.005, Student’s *t*-test) between lysate reactions supplemented with additional substrate and their unsupplemented counterparts. Error bars represent the standard error of the mean (*n* = 3).

We then evaluated production of these compounds in cell-free reactions derived from *S. venezuelae* and *S. lividans*. Time-course absorbance measurements at 340 and 318 nm captured immediate (within 1–1.5 h) flaviolin and albonoursin production, respectively (Figure 4E,F). These early spikes in metabolite synthesis are likely due to the early onset of protein translation in these *Streptomyces* extracts, as seen in our sfGFP expression data (Figure 3B). For flaviolin, production stabilizes in the *S. lividans* extract without precursor supplementation, indicating sufficient native malonyl-CoA pools in this extract to supply flaviolin synthesis (Figure 4E). On the other hand, this product falls out of detectable levels by the second hour in *S. venezuelae*, which may be attributed to a combination of low malonyl-CoA pools and the rereduction of this quinone (Figure 4E). Running the reactions in 15 mL vessels, which likely boosted protein synthesis, improved flaviolin production significantly in the *S. lividans* extract (Figure 4G). However, the *S. venezuelae* lysate required supplementation with exogenous malonyl-CoA to achieve flaviolin synthesis in a 12 h reaction, confirming a more limited endogenous supply of this precursor in this lysate (Figure 4G). For albonoursin, absorbance measurements slightly increased when the reactions were aerated (Figure 4H). However, adding more l-Phe and l-Leu to the reactions had no detectable impact on albonoursin yield, implying sufficient availability of these amino acids from the energy mix and/or the lysate (Figure 4H). Importantly, the increase in flaviolin and albonoursin signals in the 15 mL vessel reactions helps rule out the possibility that previously observed yield improvements in sfGFP expression upon aeration are specific solely to sfGFP due to, for example, GFP maturation artifacts.

Altogether, these results demonstrate the functional compatibility of our *Streptomyces* platforms with iterative and multienzyme biosynthesis, and further highlight strain-specific metabolic contexts that impact secondary metabolite synthesis *in vitro*. With this information, we decided to compare the different extracts for their ability to produce a functional, multidomain T1PK*S.*

### Comparison of T1PKS Production and Activity across *S. lividans*, *S. venezuelae*, and *E. coli* Lysates

Polyketides are a structurally diverse class of natural products with wide-ranging therapeutic and industrial applications, many of which are synthesized by iterative and modular T1PKSs.^11^ These large, multidomain megasynthases are often found from the high-GC genomes of actinomycetes such as *Streptomyces*, and are organized into modules that carry out discrete chain extension and modification steps.^9,11,71^ Their modular architecture has long made them attractive targets for rational engineering, offering a plug-and-play framework for building new-to-nature compounds.^14,72–74^ In practice, however, reprogramming the domain compositions and modular architectures of T1PKSs is challenged by elusive rules governing interdomain compatibility, module communication, and overall enzyme folding among others.^14^ Consequently, engineering biosynthetic pathways to produce modified compounds usually involves testing multiple designs of the same T1PKS pathway. However, cellular expression is tedious especially in *Streptomyces*, the source of common industrial production strains for polyketide synthesis,^42–44^ making this an impractical strategy to test a number of designs.

Establishing a cell-free expression platform for T1PKS expression and catalysis can accelerate engineering efforts. To assess which of our extracts best supports T1PKS activity, we compared the *S. lividans*, *S. venezuelae*, and *E. coli* lysates for cell-free production of an ethyl ketone, 2-methyl-3-pentanone, catalyzed by a previously engineered T1PKS (250 kDa in size), hereafter referred to as “EPKS”.^73^ The EPKS was generated by fusing LipPks1, a subunit of the lipomycin synthase from *Streptomyces aureofaciens* Tü117, with the thioesterase (TE) domain from the erythromycin PKS of *Saccharopolyspora erythraea*, and by inactivating the ketoreductase (KR) domain of LipPks1. This enzyme forms the ethyl ketone by accepting isobutyryl-CoA as a starter unit and condensing it with methylmalonyl-CoA via a ketosynthase-catalyzed reaction. This megasynthase has been previously produced from plasmids in *Streptomyces* cells, and reportedly required 9–10 days for transformation, growth and production.^73^ The EPKS sequence was cloned into the pTU1-A-SP44 vector to generate pTU1-*epks* and it was used in the CFE reactions.

The *Streptomyces*-optimized set of reaction conditions were initially used across extracts to enable direct comparison, except *E. coli* reactions were supplemented with a purified PPTase expressed from the sfp gene since these extracts do not carry the PPTase endogenously.^35^ All reactions were incubated for 8 h to allow TX-TL, after which they were supplemented with 0.17 mM each of isobutyryl-CoA, methylmalonyl-CoA, and CoA, then incubated for an additional 16 h for catalysis. To quantify the product from lysates within a broad concentration range, the ethyl ketone was derivatized to a hydrazone for improved stability and detectability.^75^ Using the 2-methyl-3-pentanone standard, an LC-MS signal with the theoretical *m*/*z* value of the derivatized product was detected and its signal level changed in proportion to the standard used within a μg/L to mg/L range, indicating derivatization of the ketone product with sufficient efficiency (Supplemental Figure 5).

**Figure 5:**
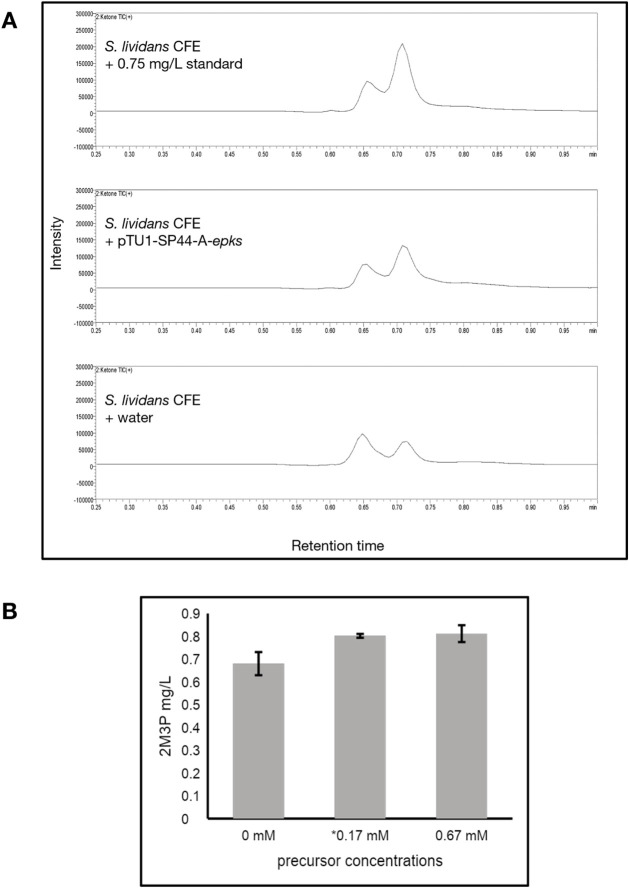
Detection of 2-methyl-3-pentanone in *S. lividans* TK24 lysate-based CFE reactions. (A) Exact ion chromatograms (EICs) of the hydrazone derivative of 2-methyl-3-pentanone ([M+K]^+^ *m*/*z*: 386.13). Chromatograms represent reactions prepared with the ketone standard, pTU1-*epks*, or water as a negative control. All reactions were derivatized with dansyl hydrazine and acetic acid. The peak corresponding to the hydrazone has a retention time of 0.702 min, verified by a standard curve (Supplemental). (B) Comparison of reactions supplemented with 0 mM, 0.17 mM, or 0.67 mM of each precursor (isobutyryl-CoA, methylmalonyl-CoA, and CoA). The previous reactions represented in (A) were prepared with 0.17 mM of each precursor. Error bars represent the standard error of the mean (*n* = 3).

We found that the ethyl ketone was produced in the *S. lividans* extract, even under the minimally optimized conditions, but not in the *S. venezuelae* or *E. coli* lysates (Figure 5A and Supplemental Figure 6). We then tested whether the *E. coli* extract itself is limiting the synthesis of the ethyl ketone. We used well-established conditions tailored toward the *E. coli* CFE (i.e., using the PANOx-SP mix and the pJL1 vector carrying a T7 promoter) and still did not detect the ethyl ketone (Supplemental Figure 6). Production still occurs without adding isobutyryl-CoA and methylmalonyl-CoA to the *S. lividans* reactions and does not improve when higher concentrations are utilized, suggesting that these precursors are readily available in the lysate (Figure 5B). These results align with the well-established use of *S. lividans* TK24 as a versatile host for polyketide synthesis, supporting its continued use as a chassis for CFE systems targeting T1PKS expression.^43,76–79^ While further optimization of TX-TL is likely to enhance metabolite production, the platform, combined with our technique to derivatize the ethyl ketone, enables production of up to 0.8 mg/L of the ethyl ketone. This workflow is therefore already suitable for evaluating titer changes from sequence variations, enabling future efforts to elucidate T1PKS engineering design rules.

## Conclusions

Through this study, we have established and validated *S. venezuelae* NRRL B-65422 and *S. lividans* TK24 lysate-based CFE systems for natural product synthesis. In doing so, we build upon foundational work in *Streptomyces* CFE while addressing critical limitations in previously published systems, particularly regarding their reproducibility, scalability, and generalizability. This work uniquely compares metabolic product formation from enzymes produced and activated in *Streptomyces* versus *E. coli* lysates and establishes the superiority of an *S. lividans* extract for the one-pot expression and catalysis of a large T1PK*S.*

Our findings highlight key discrepancies between earlier protocols and our results, underscoring the challenges of translating published methods across strains and laboratory settings. Despite genetic similarities, the reported minimal energy mix optimized for *S. venezuelae* ATCC 10712 failed to support protein production in our NRRL B-65422 strain. In addition, Li et al.’s approach using *S. lividans* B-12275 relied on purified protein additives. In contrast, we achieved >180 μg/mL protein yields in both the *S. venezuelae* and *S. lividans* strains tested here using conditions that exclude such supplements, effectively enabling simpler and more scalable natural product discovery and engineering workflows. We also demonstrate the value of aeration as an essential factor for optimizing *Streptomyces*-based TX-TL reactions. These results expand the understanding of physicochemical parameters critical for actinomycete-based CFE and should inform future lysate preparation and reaction protocols.

Our conditions do not only enable expression across lysates derived from these two *Streptomyces* strains, but across *E. coli*, *S. erythraea*, and *R. jostii* extracts as well. These conditions can thus be further improved for testing expression in the robust *E. coli* extract against various actinomycete environments in parallel and implemented to address a major challenge in modern natural discovery pipelines. In current automated workflows for large-scale BGC expression, BGC-carrying constructs are typically transformed into a single heterologous host despite host-BGC incompatibility being a major concern.^20,80^ Success rates of secondary metabolite production and detection increase when deploying several heterologous hosts, especially if the host system is closely related to the BGC’s source organism,^81,82^ but varying host-specific transformation or expression time scales complicate testing different chassis in parallel, even with automation.^10^ On the other hand, lysate-based CFE systems, regardless of the source organism, do not require transformation and can produce protein within hours. Further, BGCs may need to be cloned into different constructs for different bacteria, whereas we demonstrate that our suite of actinomycete and *E. coli* extracts can express genes from the pTU1-A-SP44 vector. We aim to further optimize this generalizable set of conditions to enable the automated parallel BGC expression across a versatile range of actinomycetes for accelerated natural product discovery.

Notably, we report the first known case of biosynthesis from a functional T1PKS expressed in a bacterial cell-free system. Following our optimized protocol, our *S. lividans* TK24 lysate was able to express an active, 250 kDa T1PKS and catalyze the formation of its ethyl ketone product in a single pot, without precursor or PPTase supplementation. These findings represent a significant step forward, as previous efforts using *E. coli* lysates were limited to a truncated T1PKS module and required PPTase coexpression, with no clear evidence of catalytic activity.^27,39^ By comparing T1PKS expression in this extract to *E. coli*, we reinforce previous findings that *Streptomyces*-based CFE systems confer advantages for natural product synthesis. Simultaneously, we demonstrate that the *S. venezuelae* system could not synthesize the T1PKS, justifying the exploration and optimization of diverse actinomycete extracts in the future. It would be important to identify components that make certain organisms and their lysates better production environments in the future to enable more robust cell-free expression systems. Notably, the current *S. lividans*-based system, in combination with a rapid and sensitive ethyl ketone detection method, can be deployed as a platform for screening variants of this T1PKS with high-throughput. Doing so can elucidate design rules in T1PKS engineering that relate to module compatibility, linker regions, and interdomain communication, enabling machine learning-guided designs.

## Methods

### Strains and Plasmids

The strains used in this study are listed in the Supporting Information. The pTU1-A-SP44-*sfgfp* plasmid was a gift from Paul Freemont and Simon Moore (Addgene plasmid #163757; http://n2t.net/addgene:163757; RRID:Addgene_163757).^47^ The sfGFP sequence in this construct is codon optimized for *Streptomyces* expression. The pJL1 plasmid, used for cell-free expression, was a gift from Michael Jewett (Addgene plasmid #69496; http://n2t.net/addgene:69496; RRID:Addgene_69496).^83^ All PCRs were performed using the KOD One PCR Master Mix (Sigma-Aldrich, KMM101). Constructs were assembled using HiFi DNA Assembly (New England Biolabs) and transformed into *E. coli* NEB 5-alpha Competent Cells (New England Biolabs, C2987) for cloning. The native *rppA* gene^33^ and the *epks* ^73^ gene were amplified from previously constructed plasmids. The albonoursin BGC genes *albA*, *albB*, and *albC* were individually amplified from a construct that was previously constructed at JGI. This original construct consists of the *albABC* genes that were codon balanced for synthesis using JGI’s Build Optimization Software Tools for DNA Synthesis (BOOST) (https://boost.jgi.doe.gov/) and a *Streptomyces venezuelae* codon usage table.^84^ The “balanced” setting in BOOST was applied to adjust synonymous codon usage to meet DNA synthesis constraints rather than to optimize for translation. This mode balances GC composition and minimizes repetitive or highly structured regions that can hinder synthesis and cloning, while preserving the original amino acid sequence. No manual replacement of specific rare codons (e.g., TTA) was performed. The *rppA* gene and the albonoursin BGC (*albABC*) sequences were cloned into the pTU1-A-SP44 vector. The *epks* gene was cloned into pTU1-A-SP44 and pJL1 vectors. Ribosome binding sites (RBSs) for all genes were designed using the Salis Lab’s RBS Calculator (https://salislab.net/software/design_rbs_calculator) using *Streptomyces venezuelae*, as a host organism and 10 000 au as a target translation initiation rate. RBS sequences were encoded within primers used to amplify genes or backbones for HiFi assembly. Plasmid maps for pTU1-A-SP44-*rppA*, pTU1-A-SP44-*albABC*, pTU1-A-SP44-*epks* constructs cloned here are supplied as Supplementary Files.

### Media and Buffer Preparation

YEME medium was prepared with 3 g/L yeast extract, 3 g/L malt extract, 5 g/L peptone, 10 g/L glucose, and 340 g/L sucrose which were autoclaved then supplemented with filter-sterilized 5 mM MgCl_2_·6H_2_O. GYM medium was prepared by autoclaving 4 g/L glucose, 4 g/L yeast extract, and 10 g/L malt extract in distilled water. Both media were pH adjusted to 7.2 with NaOH. Extraction buffers were prepared as follows: S30SA buffer consisted of 10 mM HEPES-KOH (pH 7.5), 10 mM MgCl_2_, and 1 M NH_4_Cl; S30SB buffer contained 50 mM HEPES-KOH (pH 7.5), 10 mM MgCl_2_, and 50 mM NH_4_Cl; and S30SC resuspension buffer was derived from S30SB with the addition of 10% (v/v) glycerol. HEPES-KOH stock solutions were prepared by dissolving 59.6 g of HEPES (238.3 g/mol) in 200 mL of water, adjusting the pH to ∼7.25 with KOH, and bringing the final volume to 250 mL before filter sterilization and aliquoting. MgCl_2_·6H_2_O and NH_4_Cl were dissolved in water to final buffer concentrations, followed by filtration and storage at 4 °C. Fresh DTT was added fresh on the day of use at 2 mM final concentration for each buffer.

### Cell Growth and Cell-Free Extract Preparation

*E. coli* extracts were prepared by growing cells in 2×YTPG media and lysing via sonication following a previously described protocol.^35^ For preparing actinomycete extracts after growth in YEME, cultures were consistently incubated at 28 °C for *S. lividans* and 30 °C for *S. venezuelae*, *S. erythraea*, and *R. jostii*. Each strain was cultivated in a 50 mL YEME medium in a 250 mL spring coil flask from a fresh spore glycerol stock at 200 rpm for 3 days. A 50 mL fresh YEME medium in a 250 mL spring coil flask was inoculated with 1% (v/v) (for *S. lividans* and *S. venezuelae*) or 4% (v/v) (for *S. erythraea* and *R. jostii*) of the 3-day culture and then incubated in the same conditions for overnight growth. After 24 h, 750 mL YEME medium in a 2.5 L Tunair baffled flask was inoculated with 1% (v/v) (for *S. lividans* and *S. venezuelae*) or 2% (v/v) (for *S. erythraea* and *R. jostii*) of the culture, then incubated with shaking at 220 rpm. Preliminary tests comparing harvest times (16, 20, and 48 h) indicated comparable extract activity between 16 and 20 h cultures and a loss of activity at 48 h (data not shown). A 20 h harvest time was therefore used for all subsequent lysate preparations. Cells were harvested by centrifugation at 7000 *g* for 30 min at 4 °C, and washed twice with ice-cold S30SA buffer and once with S30SB buffer. Cell pellets were weighed and flash-frozen in liquid nitrogen and stored at −80 °C. The same culture conditions were used when testing *S. lividans* grown in GYM media. Due to significantly faster growth of *S. venezuelae* in GYM compared to YEME, inoculum volumes and incubation times were modified. Specifically, spores were allowed to recover for 24 h in 50 mL GYM. A 50 mL seed culture was then initiated using a 2% (v/v) inoculum and incubated for 8 h, after which 750 mL GYM was inoculated with 1% (v/v) of the seed culture. On the day of lysis, cell pellets were thawed and resuspended in S30SC, initially using 0.8 mL buffer per gram of pellet weight. During lysis optimization, *S. venezuelae* lysates were prepared by sonicating 250 μL aliquots of the resuspended cells in 1.5 mL microcentrifuge tubes using 10 s on/off cycles at various energy inputs (75–300 J). After identifying 150 J per 250 μL aliquot as an optimal lysis condition and testing different resuspension volumes, all cell extracts were prepared by resuspending cells in 0.4 mL per gram of pellet weight then sonicating 2 mL with 1200 J using 10 s on/off cycles. Crude extracts were clarified by centrifugation at 16,000*g* for 30 min at 4 °C, and total protein concentrations were quantified via Bradford assay. After lysis optimizations, approximate total protein concentrations of *Streptomyces* lysates were 30 mg/mL. Aliquots of supernatants were flash-frozen in liquid nitrogen and stored at −80 °C.

### Cell-Free Expression Reactions

The SV minimal mix initially tested was prepared as previously described.^47^ The initial EC PANOx-SP mix that was first used to enable low sfGFP expression in *S. venezuelae* and *S. lividans* lysates comprised of 1.2 mM ATP, 1.2 mM GTP, 0.68 mM UTP, 0.68 mM CTP; 0.06 mM folinic acid, 0.4 mM nicotinamide adenine dinucleotide (NAD), 0.27 mM coenzyme A (CoA), 4 mM oxalic acid, 1 mM putrescine, 1.5 mM spermidine, 57.33 mM HEPES buffer (pH 7.0–7.6), 84 μg/mL tRNAs, 6 mM magnesium glutamate, 150 mM potassium glutamate, 1.67 mM each of the 20 amino acids, and 33 mM phosphoenolpyruvate (PEP). These concentrations were altered and components were replaced following sequential optimization experiements in the *S. venezuelae* extract. The final optimized mix comprised 0.6 mM ATP, 0.6 mM GTP, 0.34 mM UTP and 0.34 mM CTP; 0.06 mM folinic acid; 0.4 mM nicotinamide adenine dinucleotide (NAD), 1 mM putrescine, 1.5 mM spermidine, 57.33 mM HEPES buffer (pH 7.0–7.6), 9 mM magnesium glutamate, 150 mM potassium glutamate, 1.67 mM each of the 20 amino acids, 30 mM phosphoglyceric acid, and 5 mM glucose-6-phosphate. Before DNA concentration optimization, all reactions were started with 87 ng/μL DNA template. Later reactions were initiated with 200 ng/μL DNA. CFE reactions were assembled by combining 50% (v/v) extract, 33% (v/v) energy mix, and 17% (v/v) DNA. All reactions were incubated at 30 °C. For sfGFP expression optimization experiments in *S. venezuelae* extracts, reactions were terminated after 4 h. For secondary metabolite synthesis experiments, reactions were run for 8–16 h. For flaviolin and albonoursin production, the respective precursors were added during CFE reaction preparation. For 2-methyl-3-pentanone production, the precursors were added 8 h after the CFE reactions were started and the reactions were allowed to run for another 16 h. After which, the reactions were incubated overnight at 50 °C with an equal amount of methanol to facilitate the decarboxylation of any β-keto intermediates to 2-methyl-3-pentanone.^73^ In all cases, appropriate controls (i.e., CFE reactions lacking a DNA template) were prepared for comparison. The type of lysate used and changes to any of these conditions are described in the text. Reactions were set up in triplicate, as 10 μL volumes in wells of a hardshell 96-well PCR plate (Bio-Rad), or 30 μL volumes in 1.5 mL centrifuge tubes (Eppendorf) or 15 mL conical tubes (VWR) where stated. PCR plates were sealed with MicroAmp Clear Adhesive films (Applied Biosystems) for overnight time-course fluorescence measurements in a plate reader, or sealed with a Breathe-Easy sealing membrane (Sigma-Aldrich) and placed in a humidified chamber within a 30 °C incubator for 4 h end-point measurements. Reactions were left unagitated during incubations.

### Plate Reader Measurements

All measurements were taken using top optics on a BioTek Synergy H1 (Agilent). To capture fluorescence measurements of reactions expressing sfGFP, excitation and emission filters were set to 485 and 538 nm, respectively. sfGFP levels in reactions were quantified from relative fluorescence units using a standard curve prepared from purified sfGFP, following a previously described protocol.^85^ Flaviolin and albonoursin production were monitored in real time by measuring absorbance at 340 nm and 318 nm, respectively.

### LC-MS/MS Detection of Flaviolin and Albonoursin

After TX-TL, 30 μL CFE reactions were frozen then transferred to 2 mL tubes sealed with punctured caps. The samples were then lyophilized using a FreeZone 2.5L system (Labconco) for 1 h while the temperature and pressure were maintained at −48 °C and ∼0.133 mbar, respectively. Metabolites in each sample were then extracted with 120 μL methanol and by sonicating tubes in an ice bath for 15 min, followed by centrifugation at 7000*g* for 5 min at 10 °C. The recovered supernatants were concentrated at 35 °C in a SpeedVac SPD130DLX (Thermo Scientific) until solvent had evaporated (ca. 60 min). Pellets were resuspended with 120 μL methanol and 1 μg/mL ABMBA, centrifuged at 7000*g* for 5 min at 10 °C. The supernatants were recovered and filtered through Corning Costar Spin-X centrifuge tube filters (0.22 μM) and transferred to glass vials for LC-MS/M*S.* Flaviolin and albonoursin were detected using LC-MS/MS on C18 reverse-phase columns. Flaviolin analysis was performed on a Thermo Scientific QE-HF system, while albonoursin detection was carried out on a Thermo Scientific Exploris 120 instrument. Both instruments employed electrospray ionization (ESI) in positive mode. LC-MS settings and gradient parameters followed the standardized JGI-LBNL metabolomics method.^86^ Analyses were conducted on MZmine 3.8.0. Extracted Ion Chromatograms were first generated by searching for flaviolin ([M–H]^−^ *m*/*z* = 205.014) and albonoursin ([M+K]^+^ *m*/*z* = 295.144) with a mass tolerance of 10 ppm. The identified precursor *m*/*z* and corresponding retention time were then used to visualize the compounds’ corresponding MS/MS spectra.

### LC-MS Detection of 2-Methyl-3-Pentanone

After overnight incubation at 50 °C in methanol, lysate reactions and control reactions spiked with authentic 2-methyl-3-pentanone standard (reconstituted in methanol) were mixed with an equal volume of derivatization solution consisting of 40 mM dansyl hydrazine in methanol and 10% (v/v) acetic acid, to yield final concentrations of 20 mM dansyl hydrazine and 5% (v/v) acetic acid in the reaction mixture. The samples were then incubated at 50 °C for 1 h to facilitate derivatization to the hydrazone product, and centrifuged at 8000*g* for 5 min. The supernatants were recovered and filtered through Corning Costar Spin-X centrifuge tube filters (0.22 μM) and transferred to glass vials for LC-M*S.* Hydrazones were analyzed using a Nexera 40 Series ultrahigh-performance liquid chromatography (UHPLC) coupled with an LCMS 2050 mass spectrometer (Shimadzu, Japan). Separation was achieved on a Nexcol C18 column (50 × 2.11 mm, 1.8 μm, Shimadzu, Japan) operated at 50 °C. The mobile phase consisted of acetonitrile, methanol, and acetic acid 0.1% (10:89:1, v/v), set in isocratic mode with a flow rate of 0.3 mL/min. The total analytical time was 2 min with the target analyte eluting at 0.7 min. Mass spectrometry utilized a heated dual ion source (DUIS) operated in positive ion mode. Source parameters included a desolvation temperature of 300 °C, nebulizing gas at 3 L/min, drying gas at 5 L/min, and heating gas at 7 L/min. Mass spectra were acquired in selected ion monitoring (SIM) mode, targeting the potassium adduct [M+K]^+^ of the hydrazone at an *m*/*z* of 386.13.

## Supplementary Material









## Data Availability

The raw mass spectrometry data (MS/MS files) generated and analyzed in this study are being deposited in the MassIVE repository and will be accessible for peer review.

## Notes

Additional experimental data for cell-free lysate optimization and analytical measurements of produced compounds, sequences, reagents, and strains used, and detailed protocols for lysate preparation. Figure S1: sfGFP expression in *S. venezuelae* NRRL B-65422 and *S. lividans* TK24 extracts. Figure S2: reducing or increasing spermidine concentrations in *S. venezuelae* CFE reactions. Figure S3: sfGFP expression in *Saccharaopolyspora erythraea* and *Rhodococcus jostii* extracts. Figure S4: LC-MS/MS confirmation of flaviolin and albonoursin production in *E. coli* extracts. Figure S5:representative EICs of hydrazone derivatives of 2-methyl-3-pentanone ([M+K]^+^ *m*/*z*: 386.13). Table S1: albonoursin BGC sequences used in this study. Table S2: primers used in this study. RBS sequences are highlighted in yellow. Table S3: bacterial strains used in this study. Table S4: reagents used in this study. Supplemental methods: step-by-step optimized cell growth and extract preparation procedure for *S. lividans* TK24­(PDF)
pTU1-SP44-A-albABC.txt (TXT)
pTU1-SP44-A-epks.txt (TXT)
pTU1-SP44-A-rppA.txt (TXT)

## ⋈

Nutcracker Therapeutics, Emeryville, CA 94608, USA

## ⧓

Graduate School of Engineering Biology, Korea Advanced Institute of Science and Technology (KAIST), Daejeon 34141, Republic of Korea.

## Abbreviations

CFE: cell-free expression
TX-TL: transcription-translation
sfGFP: super folder green fluorescent protein
T1PKS: type I polyketide synthase
