# Supplementary material for: A Simple and Versatile Cell-Free Expression Method for Producing Secondary Metabolites
Source: ACS Synth Biol. 2025 Dec 25;15(1):99–113. doi: 10.1021/acssynbio.5c00497 (PMC12814521; doi:10.1021/acssynbio.5c00497)
Supplement: Supplementary file 1 [file sb5c00497_si_001.pdf]

Supporting Information for Publication

A simple and versatile cell-free expression method for producing secondary metabolites

Jaime Lorenzo N. Dinglasan[1], Namil Lee[2,3,4,$], Nam Ngoc Pham[5,6], Meghana Faltane[1], Marie Lynde[1], Katherine B. Louie[1], Sangeeta Nath[1,†], Jay D. Keasling[2,3,4,7,8,9], Hiroshi Otani[1,5], Nigel J. Mouncey[1,5,*]

1 US Department of Energy Joint Genome Institute, Lawrence Berkeley National Laboratory, Berkeley, CA, USA.
2 California Institute for Quantitative Biosciences (QB3), University of California, Berkeley, CA, USA
3 Joint BioEnergy Institute, Emeryville, CA, USA
4 Biological Systems and Engineering Division, Lawrence Berkeley National Laboratory, Berkeley, CA, USA
5 Environmental Genomics and Systems Biology Division, Lawrence Berkeley National Laboratory, Berkeley, CA, 94720 USA
6 Center for Advanced Bioenergy and Bioproducts Innovation, Lawrence Berkeley National Laboratory, Berkeley, CA 94720, United States
7 Department of Chemical & Biomolecular Engineering, University of California, Berkeley, CA, USA
8 Department of Bioengineering, University of California, Berkeley, CA, USA
9 Novo Nordisk Foundation Center for Biosustainability, Technical University of Denmark, Lyngby, Denmark
† Present address: Nutcracker Therapeutics, Emeryville, CA 94608 USA
$ Present address: Graduate School of Engineering Biology, Korea Advanced Institute of Science and Technology (KAIST), Daejeon 34141, Republic of Korea

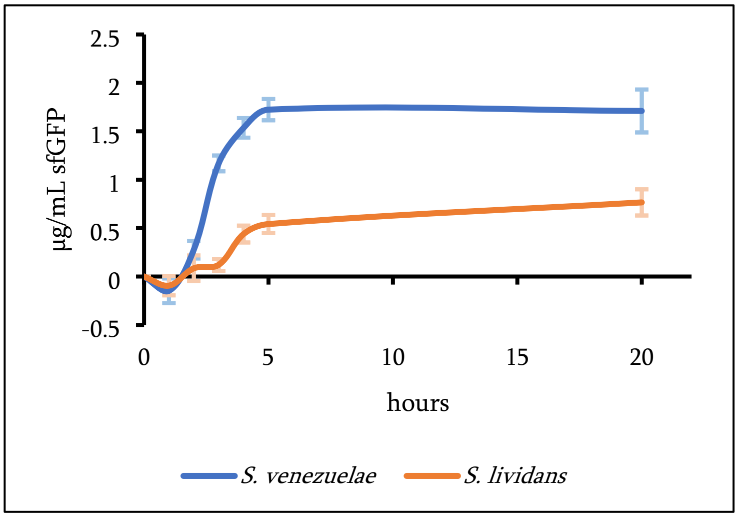

Figure S1. sfGFP expression in *S. venezuelae* NRRL B-65422 and *S. lividans* TK24 extracts, prepared using growth and lysis conditions described in[1]. Extracts were combined with the *E. coli* PANOx-SP energy mix and pTU1-A-SP44-sfGFP in 1.5 mL vessels and fluorescence readings were taken at various timepoints (n=3).

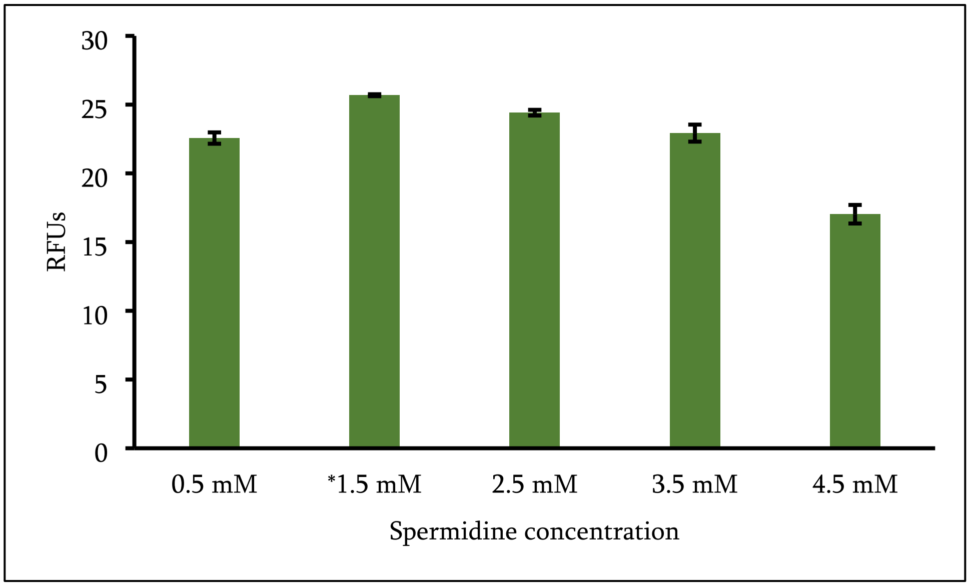

Figure S2. Reducing or increasing spermidine concentrations in *S. venezuelae* CFE reactions negatively affects sfGFP production (n=3). 1.5 mM is the concentration used throughout the manuscript.

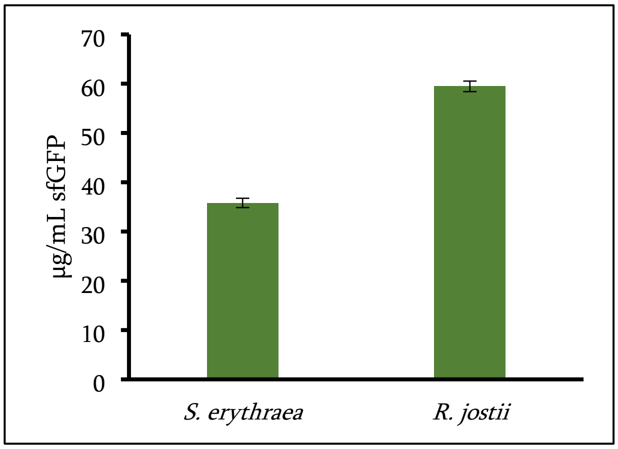

Figure S3. sfGFP expression in *Saccharaopolyspora erythraea and Rhodococcus jostii* extracts, prepared using CFE conditions optimized for *S. venezuelae* (n=3).

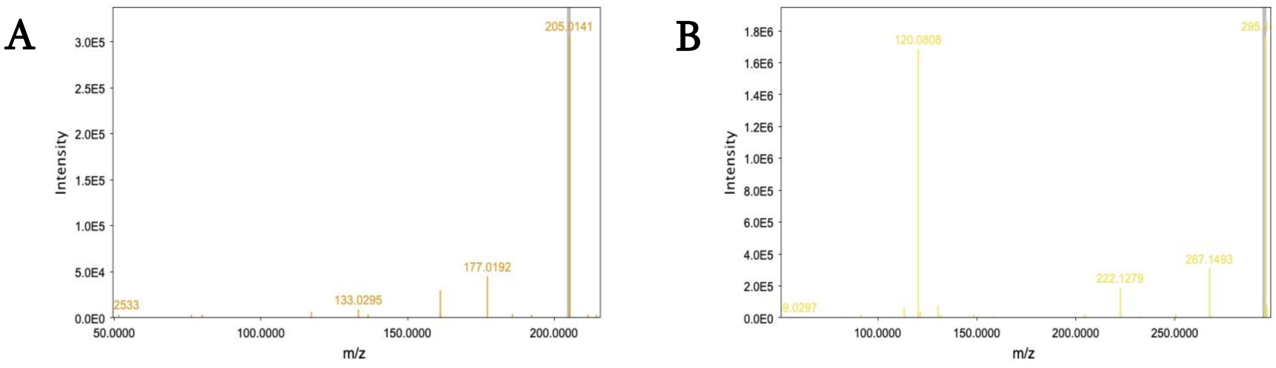

Figure S4. LC-MS/MS confirmation of flaviolin and albonoursin production in *E. coli* extracts. MS/MS spectra for A) flaviolin ([M-H]- precursor m/z = 205.014) and B) albonoursin ([M+K]+ precursor m/z = 295.14) were generated using MZmine 3.8.0.

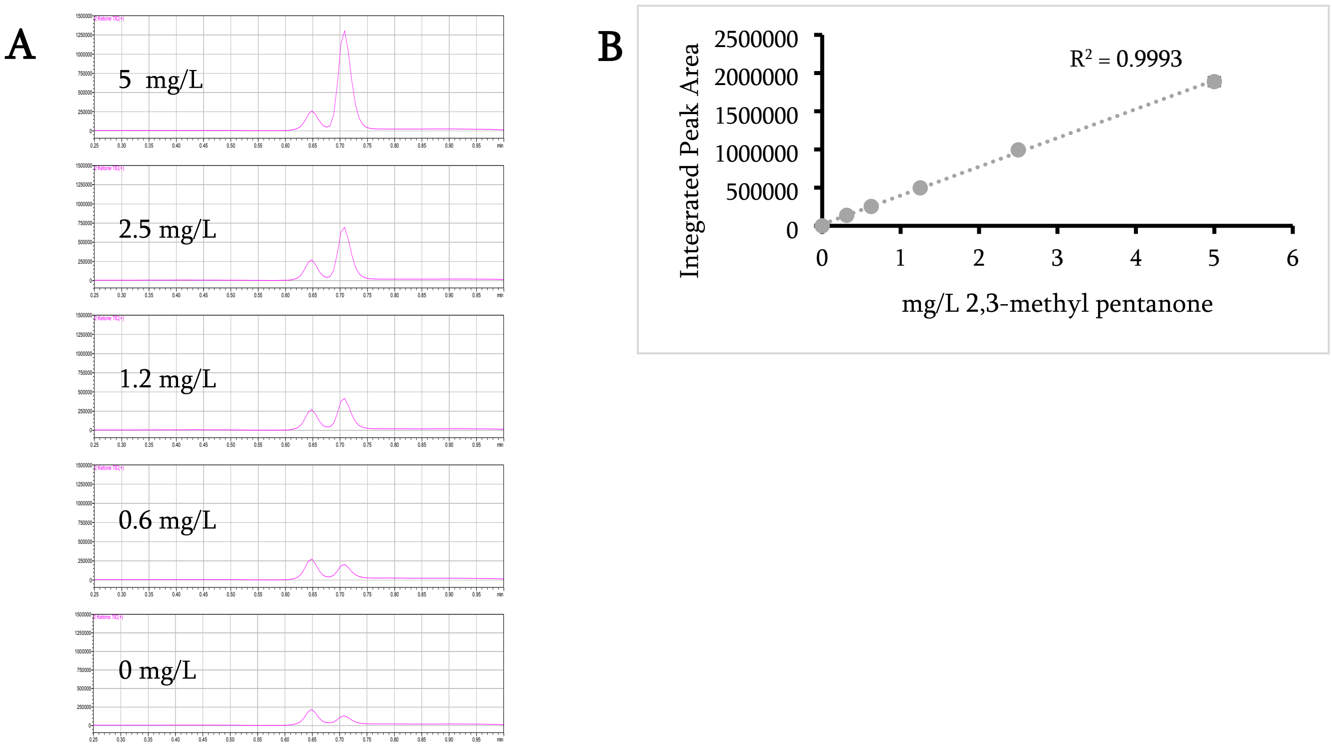

Figure S5. A) Representative EICs of hydrazone derivatives of 2,3-methyl pentanone ([M+K]+ m/z: 386.13). The 2,3-methyl pentanone standard was added in defined concentrations to mixtures of the *S. lividans* TK24 extract and optimized energy mix. These were then incubated with dansyl hydrazine and acetic acid to derivatize the ketone to the hydrazone detected here. B) Standard curve generated from integrated peak areas at retention time 0.702 min (n=3).

Table S1. Albonoursin BGC sequences used in this study.

albA	atgaggcgtcacccatcacatagtccatatcggggagggtgtgaagtaaggccaaaacgaaggggtttgatgttggcacatagtagcagtgagtcacctccagaatccttgccggacgcatggactgtacttaagaccaggactgcagtccgtaattacgcaaaggaaccagtcgatgacgcattgatcgaacagcttctggaggccatgttggcagcaccaacagcatcaaaccgtcaggcctggtcattcatggtagtacgcagaccagcagcagtgaggcgtttgcgtgcattctcacctggcgtacttggtacaccagcattcttcgtagtcgcatgcgtagaccgttctttgactgacaatttgagtcctaagttgagccagaagatatatgatacaagtaagttgtgtgtcgcaatggccgtcgaaaaccttttgttggcagcacatgcagcaggattgggtgggtgtccagtcggttctttccgatcagatatagtaacatcaatgttgggaattcctgaacacatagagccaatgttggtagtacctatagggcgaccagcaaccgcactcgtccctagtcagcgacgtgcaaagaacgaagtagtaaactacgaatcatggggaaaccgcgcggcggctccaactgcctga
albB	atgaacccaggtgagactgtattgcctccacagttgcgtgaggaaatagcattgctcgcagtgtatcttttgagttcaggtaggggtcttttggaggagccagcggattatggtatttatcggtgtacagatggcgcacgacgcgccctgcagttgttggatgaacacggtggaagtactgccagactgacagcggtacgcgagcgtttggacgaagtaatgttcgcgccaatgggagaagatagggatatgggtgcaatattggacgatctttgccgccaaatggcggacgccctgccagaaattgagacaccatga
albC	atgttggcaggattggttcctgcaccagatcatggtatgcgagaagaaatcttgggtgatcggtcaaggttgataaggcagcgaggggaacacgcactgataggtatcagtgcagggaacagttacttctcacagaaaaatacagtaatgttgctccagtgggccggtcagcgcttcgagcggacagatgtggtatacgtcgatacacacattgatgaaatgttgatcgcagacggtcgtagtgcacaggaggcagagcggagtgtaaaaaggacacttaaggacttgcgtcgacgtttgcgtcggagtttggaaagcgttggagatcacgcggaacggttccgtgtacgaagcctttcagaattgcaggaaacaccagaatatcgagcagtccgtgagcgtacagaccgtgcattcgaagaggatgcagaattcgcaaccgcatgtgaggatatggtacgggcggtagtaatgaacagaccaggtgacggagtgggtatatcagcagaacatcttcgggcaggacttaactatgtacttgcggaggcaccattgttcgcagattcgccaggggtattcagtgtaccatcatccgtattgtgctatcacatagatacaccaatcacagcattcctttcacgacgtgagacgggattccgtgcagcagagggtcaagcgtatgtagtggtgcgacctcaggaattggcagacgcagcatga

Table S2. Primers used in this study. RBS sequences are highlighted in yellow.

pTU1 backbone forward for *rppA* and *albABC*	ggatcctcgaccaggcatc
pTU1 backbone reverse for *rppA* and *albABC*	tacaccagactttacaacaccgc
rppA forward	gcggtgttgtaaagtctggtgtagtactttaactttaagaaggagatatacatatggcgaccctgtgccgac
rppA reverse	tatttgatgcctggtcgaggatccttatttttcgaactgcgggtggctcc
albA forward	gtgttgtaaagtctggtgtaataagtatagagtactatcaaataagtttatttaaatgaggcgtcacccatcacatag
albA reverse	ggggtacctagtatatagtagttattacttactcaggcagttggagccgc
albB forward	tgcctgagtaagtaataactactatatactaggtaccccatgaacccaggtgagactgtattgc
albB reverse	taaggacctcctctgactctctgtatatgagtcatggtgtctcaatttctggcagg
albC forward	tgactcatatacagagagtcagaggaggtccttaatgttggcaggattggttcctg
albC reverse	gaattggcagacgcagcatgaggatcctcgaccaggcatcaa
pTU1 backbone forward for *epks*	cgtcacattgacgcctggctgggtggcggcaacagctgaggatcctcgaccaggcatc
pTU1 backbone reverse for *epks*	cgatgttcagacataaaataccttttgccagtgtagattgagggtacaccagactttacaacaccgcac

pJL1 backbone forward for *epks*	cgtcacattgacgcctggctgggtggcggcaacagctgagtcgaccggctgctaacaaag
pJL1 backbone reverse for *epks*	cgatgttcagacataaaatacctttgccagtgtagattgagggattatttctagagggaaaccgttgtggtc
epks forward	ccctcaatctacactggcaaaggtattttatgtctgaacatcgcggtagcg
epks reverse	tcagctgttgccgccac

Table S3. Bacterial strains used in this study.

**Organism**	**Strain**	**Source**
Escherichia coli	BL21 Star (DE3)	New England Biolabs
Streptomyces venezuelae	NRRL-B65422	[2]
Streptomyces lividans	TK24	[3]
Saccharopolyspora erythraea	RHA1	[4]
Rhodococcus jostii	NRRL-2338	[5]

Table S4. Reagents used in this study.

**Reagent**	**Catalog**
NaCl	S9888
Tryptone	T9410-250G
Yeast Extract	Y1625-250G
Potassium Phosphate Dibasic	P3786-500G
Potassium Phosphate Monobasic	P5655-100G
D-Glucose	G8270-100G
Tris Base	T6066-100g
Magnesium Acetate	M5661-250G
Potassium Acetate	P1190-500G
DTT	R0861
ATP	FERR0181
GTP	FERR0181
CTP	FERR0181
UTP	FERR0181
Folinic Acid	47612-250mg
tRNA	1.01E+10
NAD	N8285
CoA	C4780-10MG
Oxalic Acid	241172-50g
Putrescine	51799-100mg
Spermidine	S2626-1G
HEPES Solution (pH 7.0-7.6)	H0887-20mL
Magnesium Glutamate	49605-250G
Ammonium Glutamate	FG28929
Potassium Glutamate	G1501-100G

L-Valine	V0513-25G
L-Tryptophan	T0254-25G
L-Phenylalanine	P5482-25G
L-Isoleucine	I2752-25G
L-Leucine	L8000-25G
L-Cysteine	C7352-25G
L-Methionine	M5308-25G
L-Alanine	A7469-25G
L-Arginine	A8094-25G
L-Asparagine	A0884-25G
L-Aspartic Acid	A9256-100g
L-Glutamic Acid	G1251-100
Glycine	G7126-100g
L-Glutamine	G8540-25G
L-Histidine	H8000-25G
L-Lysine	L5501-25G
L-Proline	P0380-100g
L-Serine	S4311-25g
L-Threonine	T8625-10g
L-Tyrosine	T8566-25G
PEP	10108294001
3-Phosphoglyceric Acid	ADVH9B9B5C8F
Glucose-6-Phosphate	10127647001

Supplemental Methods. A step-by-step protocol for the preparation of CFE reactions from *Streptomyces lividans* TK24 cells using the optimized conditions described in the manuscript.

<u>Media and Buffer Preparation</u>

1. Prepare 1 L YEME medium.
   1.1. In a 2 L bottle, combine:
      1.1.1. 3 g yeast extract
      1.1.2. 3 g malt extract
      1.1.3. 5 g peptone
      1.1.4. 10 g glucose
      1.1.5. 340 g sucrose
      1.1.6. 950 mL distilled H_2O.
   1.2. Adjust to pH 7.2 using 5 M NaOH.
   1.3. Autoclave at 121 °C for 20 min.
   1.4. Separately, dissolve 510 mg $MgCl_2{\cdot}6H_2O$ in 50 mL distilled H_2O and filter sterilize.
   1.5. Pour $MgCl_2{\cdot}6H_2O$ solution into autoclaved solution once cooled.
2. Prepare 500 mL S30SA buffer.
   2.1. To an autoclaved bottle, add:
      2.1.1. 5 mL 1 M HEPES solution (pH 7.0-7.6)
      2.1.2. 1.02 g $MgCl_2{\cdot}6H_2O$ (MW = 203.3 g mol^{-1})
      2.1.3. 26.98 g NH_4Cl (MW = 53.49 g mol^{-1})

        2.1.4. Add distilled H_2O to 499 mL total volume.
        2.1.5. Filter sterilize.
        2.1.6. Add 2 mM DTT on the day of use: 1 mL of 1 M DTT per 500 mL buffer.
        2.1.7. Keep ice-cold until used for cell washing.

3. Prepare 250 mL S30SB Buffer
    3.1. To an autoclaved bottle, add:
        3.1.1. 12.5 mL 1 M HEPES–KOH (pH 7.5)
        3.1.2. 508 mg $MgCl_2 \cdot 6H_2O$
        3.1.3. 674 mg NH_4Cl
        3.1.4. Add distilled H_2O to 250 mL total volume.
        3.1.5. Filter sterilize.
        3.1.6. Reserve 50 mL in a separate sterile bottle to prepare S30SC (below).
        3.1.7. Add 2 mM DTT to 200 mL volume of S30SB on the day of use: 0.4 mL of 1 M DTT per 200 mL S30SB buffer.
        3.1.8. Keep ice-cold until used for cell washing.
4. Prepare 5 mL S30SC Resuspension Buffer (on day of lysis)
    4.1. Aliquot 4.5 mL S30SB from 50 mL reserve to a sterile 15 mL conical tube, add:
        4.1.1. 10% (v/v) glycerol to each tube: 0.5 mL 100 % (v/v) glycerol.
        4.1.2. Add 2 mM DTT on the day of use: 10 µL of 1 M DTT per 5 mL buffer.
        4.1.3. Mix gently to avoid bubbles.
    4.2. Keep ice-cold until used for cell resuspension and lysis.

S. lividans TK24 Cell Growth and Harvest

1. Inoculate 50 mL YEME in a 250 mL spring coil flask with a fresh spore glycerol stock of *S. lividans* TK24. Incubate at 28 °C, 200 rpm, for 3 days.
2. Inoculate a fresh 50 mL YEME (250 mL spring coil flask) with 1% (v/v) of the 3-day starter culture. Incubate at 28 °C, 200 rpm, for 24 h.
3. Inoculate 750 mL YEME in an autoclaved 2.5 L Tunair baffled flask with 1% (v/v) of the overnight seed culture. Incubate at 28 °C, 220 rpm for 20 h.
4. Divide the culture into 4 250 mL Nalgene™ (Sigma-Aldrich) centrifuge bottles. Avoid transferring foam or pipette out the foam using a sterile pipette.
5. Pellet the culture by centrifugation at 7,000 × g for 30 min at 4 °C.Discard supernatant and immediately place bottles on ice.
6. In a cold room or on ice, wash each pellet by vortexing cells with ~ 30 mL ice-cold S30SA buffer until fully resuspended. Transfer cell resuspensions from each bottle to its own pre-weighed 50 mL conical tube.
7. Centrifuge the 4 conical tubes at 7,000 x g for 5 min 4 °C. Discard supernatant and immediately place tubes on ice.
8. Repeat wash step with 30 mL S30SA buffer.
9. In a cold room or on ice, wash each pellet with 30 mL S30SB buffer.
10. Centrifuge tubes at 7,000 x g for 5 min 4 °C. Completely discard supernatant and immediately place bottles on ice.
11. Dry tubes and weigh the washed pellets.
12. Flash-freeze pellets by dipping tubes in liquid nitrogen.
13. Store the frozen pellets at −80 °C until lysis.

Cell Lysis and CFE Extract Preparation.

1. On day of lysis, thaw the frozen pellet on ice for 40 min.
2. Resuspend the pellet in S30SC buffer at a ratio of 0.4 mL buffer per gram wet cell weight.
    2.1. This 0.4 mL/g resuspension ratio reflects the final optimized condition.

3. Transfer 2 mL of this resuspension into a 50 mL conical tube kept on ice.
4. Immerse the 3.2 mm diameter probe of a Q125 Sonicator (QSonica) ~ 30 % below the surface of the cell suspension.
5. Sonicate using 10 s on / 10 s off cycles until a total input energy of 1200 J per 2 mL suspension is delivered.
   5.1. It is important that the sample is kept cold throughout.
6. Aliquot 1 mL of the sample to 1.5 mL tubes then clarify by centrifugation at 16,000 × g for 30 min at 4 °C.
7. Recover the lysate, which is the translucent top layer of the centrifuged sample, using a 1 mL pipette tip. Recoverable lysate can be as little as ~ 0.5 mL.
8. Measure total protein concentration by Bradford assay.
   8.1. Typical optimized *Streptomyces* extracts yielded ~ 30 mg/mL total protein.
9. Aliquot the clarified extract.
10. Flash-freeze aliquots in liquid nitrogen.
11. Store aliquots at −80 °C.

<u>CFE reaction preparation</u>

1. Prepare the Small Molecule Master Mix (SMM) by combining the following reagents:
   1.1. 90 μL ATP (100 mM)
   1.2. 90 μL GTP (100 mM)
   1.3. 50.6 μL CTP (100 mM)
   1.4. 50.6 μL UTP (100 mM)
   1.5. 94.4 μL folinic acid (10 mM)
   1.6. 60 μL NAD (100 mM)
   1.7. 60 μL putrescine (250 mM)
   1.8. 90 μL spermidine (250 mM)
   1.9. 855 μL HEPES (1 M, pH 7.0–7.6)
   1.10. 764.3 μL molecular-grade water
   1.11. Mix gently and store aliquots at −80 °C.
2. Assemble a 30 μL CFE reaction in a tube on ice in the following order:
   2.1. 4.42 μL SMM
   2.2. 2 μL K-glutamate (2.25 M)
   2.3. 1.58 μL Mg-glutamate (182 mM)
   2.4. 1 μL G6P / 3-PGA mix (900 mM 3-PGA + 150 mM G6P)
   2.5. 1 μL amino acid mix (50 mM each)
   2.6. 15 μL crude extract
   2.7. 5.1 μL DNA
3. Mix gently by pipetting; keep on ice until incubation.
4. Incubate reactions at 30 °C for 4–16 h, depending on the target (shorter for GFP expression; longer for pathway assays).

1 Li, J., Wang, H. & Jewett, M. C. Expanding the palette of Streptomyces-based cell-free protein synthesis systems with enhanced yields. *Biochemical Engineering Journal* **130**, 29-33, doi:10.1016/J.BEJ.2017.11.013 (2018).

2 Gomez-Escribano, J. P. *et al. Streptomyces venezuelae* NRRL B-65442: genome sequence of a model strain used to study morphological differentiation in filamentous actinobacteria. *Journal of Industrial Microbiology and Biotechnology* **48**, doi:10.1093/jimb/kuab035 (2021).

3 Rückert, C. *et al.* Complete genome sequence of Streptomyces lividans TK24. *Journal of Biotechnology* **199**, 21-22, doi:10.1016/j.jbiotec.2015.02.004 (2015).

4 Oliynyk, M. *et al.* Complete genome sequence of the erythromycin-producing bacterium Saccharopolyspora erythraea NRRL23338. *Nature Biotechnology* **25**, 447-453, doi:10.1038/nbt1297 (2007).

5 McLeod, M. P. *et al.* The complete genome of Rhodococcus sp. RHA1 provides insights into a catabolic powerhouse. *Proceedings of the National Academy of Sciences* **103**, 15582-15587, doi:10.1073/pnas.0607048103 (2006).
